# Retroperitoneal laparoscopic nephroureterectomy with distal and intramural ureter resection for a tuberculous non - functional kidney

**DOI:** 10.1590/S1677-5538.IBJU.2017.0326

**Published:** 2018

**Authors:** Canqiang Li, Yi Yang, Le Xu, Minjie Qiu

**Affiliations:** 1 Department of Urology, The Affiliated Hexian Memorial Hospital of Southern Medical University, Guangzhou, Guangdong, China

**Keywords:** Tuberculosis, Renal, Nephroureterectomy, Nephrectomy

## Abstract

**Objective:**

To evaluate the safety and feasibility of total retroperitoneal laparoscopic nephroureterectomy with urinary-bladder junction resection for a tuberculous nonfunctional kidney.

**Materials and Methods:**

A total of 27 individuals diagnosed with unilateral nonfunctional kidney secondary to tuberculosis were treated between June 2011 and June 2015. All patients had normal renal function on the contralateral side and underwent the standard four-drug anti-tuberculosis treatment for at least four weeks before surgery. Total retroperitoneal laparoscopic nephroureterectomy was performed in all patients, and the urinary-bladder junction of distal ureter was managed using different auto-suture techniques.

**Results:**

Nineteen male and 8 female patients with an average age of 47.3 years (range, 36-64 years) underwent surgery. All the operations were successfully performed without conversion. The median operative time was 109.3 min (range, 75-138 min), the median blood loss was 157.5 mL (range, 70-250 mL), and the median hospitalization time was 3.7 days (range, 3-6 days). No serious perioperative complications occurred. Anti-tuberculosis chemotherapy was prescribed to all patients, with the entire course of treatment lasting six months. No recurrence of tuberculosis of the bladder or the contralateral kidney was observed during the median follow-up period of 26.7 months (range, 6-54 months).

**Conclusion:**

Retroperitoneal laparoscopic nephroureterectomy with urinary-bladder junction resection is a safe and feasible approach for the treatment of tuberculous non-functional kidneys.

## INTRODUCTION

Tuberculosis (TB) remains a major challenge to human health worldwide. In 2013, 9 million people fell ill with TB, and 1.5 million died from the disease (1). The kidneys are a common site of extrapulmonary TB, and the incidence of renal TB is estimated to be up to 73% in TB patients from regions with an extremely high prevalence of pulmonary TB (2). Because of its slow progress and nonspecific manifestations, the diagnosis of renal TB is often delayed, by which point one of the kidneys may have stopped functioning. The condition usually results from extensive calcification of the renal parenchyma and/or multiple infundibular stenosis or ureteric strictures. Therefore, the surgical management of nephrectomy, whether using a traditional open approach or a retroperitoneoscopic approach, becomes inevitable for those patients with a non-functional kidney. However, it remains controversial whether total resection of the ipsilateral ureter is safe and feasible during the performance of retroperitoneal laparoscopic nephrectomy in tuberculous patients. Although total resection of an affected ureter is rarely indicated for the treatment of a tuberculous non-functional kidney, the removal of the more tuberculous tissues associated with an affected ureter could theoretically prevent the occurrence of ureteral stump syndrome. This syndrome is clinically interpreted as febrile urinary tract infections, hematuria, and lower quadrant pain that may occasionally occur when a ureteral stump, a segment of the ureter, is left in place after nephrectomy. However, to the best of our knowledge, few studies have reported on the feasibility and outcome of retroperitoneal laparoscopic nephroureterectomy for the treatment of tuberculous non-functional kidneys (3, 4).

In this present study, we report our experience with retroperitoneal laparoscopic nephrou-reterectomy for the treatment of tuberculous non-functional kidneys, with the aim of evaluating the feasibility and safety of this approach.

## PATIENTS AND METHODS

Institutional review board approval was obtained from the ethics committees prior to the initiation of the study. Information from a database of prospectively collected data that included the hospital chart data and complications of all patients treated with total retroperitoneal laparoscopic nephroureterectomy was retrospectively reviewed. The diagnosis of a tuberculous non-functional kidney was established based on clinical manifestations, urinalysis, real-time polymerase chain reaction (PCR) for Mycobacterium tuberculosis, the erythrocyte sedimentation rate (ESR), intravenous pyelography, enhanced computed tomography and a nephrogram. In certain patients, cystoscopy with a bladder biopsy was performed when the clinical diagnosis of TB was not confirmed. The glomerular filtration rate of the kidney was also evaluated via a renal nuclear scan. A unilateral non-functional kidney was defined by a glomerular filtration rate of the diseased kidney of less than 15 mL/min/1.73 m^2^, associated with a cortical thickness of less than 5 mm, whereas the glomerular filtration rate of the contralateral side was normal or more than 60 mL/min/1.73 m^2^. Patients were excluded from the study if they had a history of retroperitoneal surgery on the ipsilateral side of the diseased kidney or if assessment of the presence of renal TB was not performed. Patients in the active phase of TB were also excluded.

### Study population

In all, 27 patients with a unilateral non-functional kidney due to TB who were admitted to our hospital between June 2011 and June 2015 were included in this study. Information regarding the advantages and risks of the laparoscopic surgery was provided for formal informed consent, and permission was obtained from the patients before surgery. The group included 19 males and 8 females, and the tuberculous lesion occurred on the right side in 16 patients and the left side in 11 patients. The mean age was 47.3 years (range, 36-64 years). The most common clinical manifestation was irritative voiding symptoms (19 cases), followed by recurrent urinary tract infection (5 cases), gross hematuria (2 cases) and ipsilateral flank pain (1 case). Standard 4-drug anti-TB chemotherapy, including isoniazid 10 mg/kg, rifampicin 10 mg/kg, pyrazinamide 20 mg/kg and ethambutol 15 mg/kg, was prescribed to every patient for simultaneous intake once daily for at least 4 weeks before the operation.

### Operative technique

**Figure 1:**
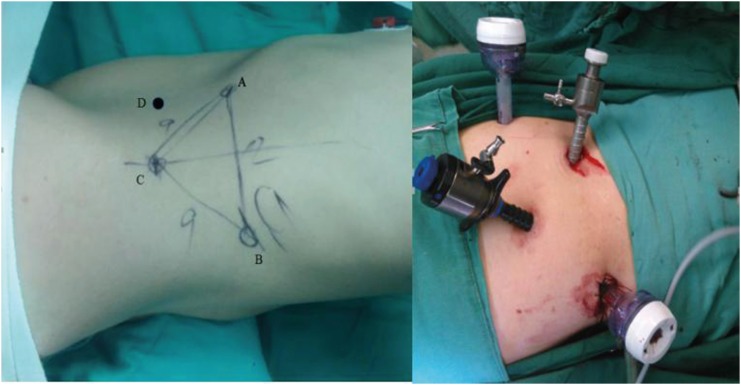
Distribution of the 4 port sites (Port D was established after nephrectomy).

All the operations were performed by a surgeon who had mastered the technique of retroperitoneoscopic radical nephrectomy. After general anesthesia induction, the patients were placed in the lateral flank position with elevation of the waist on the surgical side. Three laparoscopic working channels were first established to perform the nephrectomy approach and the dissection of the upper ureter (Figure-1). A 1.5 cm skin incision was made 2 cm above the intersection point of the axillary line and the iliac crest (port C). The muscle layer and lumbodorsal fascia were bluntly penetrated and distracted with forceps. Dilation of the retroperitoneal space was performed using a homemade balloon (by tying the finger of a glove over an F10 red rubber catheter) inflated with air to a volume of up to 500700 mL (5). A 10 mm trocar was placed at port C to form the laparoscope working channel, and pneumo-retroperitoneum was created by carbon dioxide insufflation, with its pressure maintained at 15 mm Hg. A 10 mm trocar and a 12 mm trocar were inserted under the monitoring laparoscope at the anterior axillary line around the 11th rib tip (port A) and the posterior axillary line around the 12th rib (port B), respectively. Gerota's fascia was incised, and the renal hilum was explored first by separating the space between Gerota's fascia and the psoas fascia. The renal pedicle was sought from bottom to top along the plane behind the ureter on the left side or in front of the inferior vena cava on the right side. After the vessels in the renal hilum were clearly identified, three Hem- -O-Lock clips (Teleflex Medical, Research Triangle Park, NC, USA) were utilized to ligate the renal artery and vein. That procedure had to be accomplished before dissecting the diseased kidney from the surrounding tissue to reduce bleeding. The remaining dissection of the kidney was performed in the space between Gerota's fascia and the fatty capsule of the kidney. Adhesions and scarring were usually present during the isolation of the ventral side of the tuberculous kidney, but it was not too difficult to separate. The main reason for dissection within Gerota's fascia was to reduce the prospect of peritoneal injury. The adrenals were left in situ during the isolation of the upper renal pole in all patients. The proximal ureter was identified during the dissection of the lower pole of the kidney and ligated using one Hem-O-Lock clip in case tuberculous urine leaked from the kidney collecting system. The ureter was separated distally from the bifurcation of the common iliac artery.

**Figure 2:**
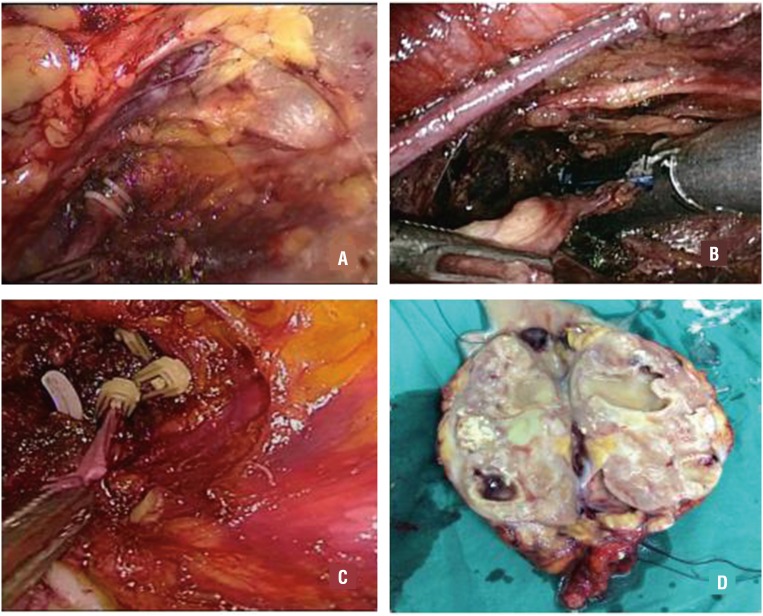
The different methods of mutilation of the distal ureter. (A: the Hem-O-Lock clip; B: Endo-GIA; C: The absorbable Lapro-Clip and D; the renal specimen).

Port D was established in the midclavicular line under the monitoring laparoscope and formed an isosceles triangle together with ports A and C. The position of the patient was then changed through adjustment of the operating Table to attain a head-down posture. The surgeon also changed his standing position from the back side of the patient to the ventral side. The laparoscope was inserted in port A, and ports B and D were the main working channels for dissection of the lower ureter. In premenopausal female patients, the uterine artery was preserved during isolation of the pelvic segment of the ureter. A grasper was inserted through port C to stretch the ureter toward the head side. The distal ureter was dissected toward the bladder until the enlargement of the intramural ureter appeared. The sectioning of the ureteral enlargement was performed using a Hem-O-Lock clip, a 30 mm Endo-GIA (Tyco Healthcare Group LP, Glover Avenue, CT, USA) or an absorbable 12 mm Lapro-Clip (Covidien IIC, Hampshire Street, MA, USA) (Figure-2). An extending incision was then made for port A, through which the tuberculous kidney and the entire ureter were placed in a surgical bag and removed together.

Finally, drainage tubes were placed through ports C and D in the retroperitoneal space near the renal pedicle and the bladder wound, after which the trocar incisions were closed.

## RESULTS

All operations were successfully performed without conversion to open surgery. The median operation time was 109.3 min (range, 75-138 min), and the median blood loss was 157.5 mL (range, 70-250 mL) without intraoperative transfusion of blood cells. The terminal ureters of 5 patients and 3 patients were ligated with Hem-O-Lock clips and Endo-GIA, respectively. Ligation of the terminal ureter in the remaining 19 patients was accomplished with an absorbable Lapro-Clip. The median weight of the tuberculous kidneys was 141.8 g (range, 25-527 g). The perioperative complications are shown in Table-1. No injury to the inferior vena cava or abdominal aorta occurred, and there was no injury to adjacent abdominal organs. Moreover, acute renal failure did not occur in any patient after surgery. There were 2 patients and 1 patient who experienced injury to the renal vein and lumbar vein, respectively, mainly resulting from adhesions around the renal hilum. Rupture of the distal ureter occurred in 1 patient during dissection of the lower ureter, probably because of the increased fragility of the diseased ureter and traction from the grasper. No spillage of pus occurred in that patient because the upper ureter had been ligated with a Hem-O-Lock clip in advance. One patient experienced the complication of incision infection, and the infection wound healed after dressing and the application of antibiotic. Four patients experienced pneumoscrotum that generally disappeared completely without any intervention within three days after surgery. Three patients experienced postoperative pyrexia. Their maximum body temperature was not over 39°C, and they were all cured with conservative therapy. In all, the median hospitalization time was 3.7 days (range, 3-6 days). Pathological examination confirmed the preoperative diagnosis of renal TB in all patients, and the pathological characteristics of TB were found in the diseased kidney and ureter. Anti-TB chemotherapy was continually performed in all patients after surgery, with a total treatment period of 6 months. During the median follow-up period of 26.7 months (range, 6-54 months), the signs of irritation of the bladder disappeared, and routine urine tests and tests for acid-fast bacilli in the urine became negative. All patients were scheduled to undergo ultrasonography of the urinary tract at 3 months, 6 months and 12 months after the operation. Cystoscopic examination was not carried out routinely after surgery. Two patients who complained of notably frequent micturition and urgency underwent cystoscopy. Bladder tumors and stones did not occur, and the symptoms were relieved by α_1_-blocker treatment. No recurrence of TB of the bladder or the contralateral kidney was observed during the follow-up period.

**Table 1:** Details of perioperative complications.

Complications		Number	Percentage
**Intraoperative**		5	18.5%
	Peritoneum injury	1	3.7%
	Renal vein injury	2	7.4%
	Lumbar vein injury	1	3.7%
	Rupture of distal ureter	1	3.7%
**Postoperative (Clavien Grade)**		**8**	**29.6%**
	Fever (Grade I)	3	11.1%
	Pneumoscrotum (Grade I)	4	14.8%
	Incision infection (Grade II)	1	3.7%

## DISCUSSION

TB is still a major challenge to public health in developing countries (6). China has the second greatest number of cases of TB in the world, with 1.3 million new cases arising per year (7). The urogenital system is the second most common site of extrapulmonary TB after lymphatic TB, occurring in 4-73% of adult TB patients (2). Because of its insidious onset and nonspecific symptoms, which mostly manifest as irritation of the bladder, late diagnosis of urinary system TB is quite common, especially in developing countries. A non-functional kidney due to destruction of the renal collecting system by Mycobacterium tuberculosis is usually present in patients with a late diagnosis. Anti-TB drug therapy has been available for tuberculous patients since the 1950s and remains the major first-line therapeutic regimen. However, this treatment is not able to cure patients with a late diagnosis. Nephrectomy intervention is recommended when there is a non-functional kidney, whether the diseased kidney is associated with calcification or is extensively destroyed, accompanied by hypertension or ureteropelvic junction obstruction (8). Figueiredo et al. reported that the rate of nephrectomy among 8961 cases of urogenital TB was 27.6% and that the difference in the nephrectomy rate between developed and developing countries was not significant (27.9% vs. 26.0%) (9). Multiple studies have shown that the retroperitoneal laparoscopic procedure is an optimal alternative to open surgery for treating patients with a non-functional kidney resulting from TB (5, 10, 11). This procedure not only reduces interference with the abdominal organs but also avoids the possible spread of TB bacteria into the abdominal cavity. However, this approach remains a challenge for surgeons in certain cases because of perinephric adhesion and poor anatomic landmarks.

The main reasons for the conversion of retroperitoneoscopic resection of the tuberculous kidney to open surgery are major bleeding and difficulty in separating perinephric adhesions (5, 12). In the present study, the renal pedicle was controlled early, before dissecting the tuberculous kidney, to reduce intraoperative bleeding. Perinephric adhesions were another major challenge during the operation because the normal anatomic structure had usually been destroyed by the infection. The closer that adhesions were to the surface of the tuberculous kidney, the more severe the adhesions were. Duarte et al. reported that transperitoneal laparoscopic dissection outside of Gerota's fascia achieved a success rate of 72% in the management of patients with inflamed kidneys (13). Those authors preferred the transperitoneal approach to the retroperitoneal approach to avoid adhesions and fibrous tissue, mainly because the former allowed more work space and clear anatomic landmarks. However, the selection of the transperitoneal vs. retroperitoneal approach depends mainly on surgeon preference. In the present study, we endeavored to perform renal dissection within Gerota's fascia to reduce the risk of peritoneal injury because carbon dioxide gas would enter the abdominal cavity if injury to the peritoneum occurred. The retroperitoneal space would then be reduced to such an extent that it would impact the operation, especially during dissection of the lower ureter. Although the normal anatomic structure had been destroyed in certain severe cases, we found that the infective adhesion between Gerota's fascia and the fatty capsule was not difficult to separate.

Laparoscopic nephroureterectomy has been shown to be feasible and safe for upper-tract urothelial carcinoma (14, 15), but the practicability of this procedure has rarely been assessed in the management of patients with tuberculous non-functional kidneys. Tuberculous lesions can affect the entire collection system, including the kidney, ureter and bladder. The more tuberculous that lesions resected from a diseased ureter are, the less likely postoperative ureteral stump syndrome is to occur (3, 16). In addition, if tuberculous lesions of the distal ureter persist, contracture of the bladder and delayed healing may result. In the present study, the lower ureter was resected as distally as possible. The complete excision of bladder cuff can be achieved via transperitoneal laparoscopic surgery (17, 18); however, this is difficult to achieve using the retroperitoneal laparoscopic technique because of the limited retroperitoneal space. Furthermore, the resection of bladder cuff would reduce the bladder capacity of tuberculous patients to some extent and may have adverse effects on the recovery of postoperative bladder function.

Three devices were utilized to ligate the terminal ureter, and these exhibited different characteristics. Endo-GIA can be used to effectively close the terminal ureter, but this device is too expensive to apply in patients from developing countries, who usually do not have medical insurance. Moreover, both Endo-GIA and large Hem- -O-Lock clips are non-absorbable and may move into the bladder, resulting in stone formation and urinary infection (19-21). Therefore, absorbable Lapro-Clips have been used to ligate the distal ureter at our center since November 2012 to prevent the possibility of stone formation in the long term.

In the present study, the median operation time and blood loss were 109.3 min and 157.5 mL, respectively. Chibber et al. reported that they detached and ligated the distal ureter below the level of bifurcation of the common iliac artery and that the corresponding indicators were 208.5 min and 326.25 mL in the tuberculosis TB group (3). Tian X et al. described the use of a Gibson incision to dissect the distal ureter of patients with tuberculous non-functional kidneys, with a median operation time and blood loss of 123.0 min and 134.0 mL, respectively (4). Compared with these studies, the present study showed a shorter operation time and less blood loss. With respect to perioperative complications, no serious complications above grade III of the modified Clavien classification occurred in our study or the two cited studies, except for a retroperitoneal hematoma after surgery that required re-operation in the study by Tian X et al. (4). To prevent the potential contamination of the surgical field by tuberculous tissue, it is essential to utilize a surgical bag to completely remove the tuberculous specimen.

The present study is limited by its retrospective design, its focus on a single center and the relatively short period of follow-up. However, to the best of our knowledge, there have been no other studies to date that have included a larger sample size of patients with tuberculous non-functional kidneys who underwent total retroperitoneal laparoscopic nephroureterectomy.

## CONCLUSIONS

Total retroperitoneal laparoscopic nephroureterectomy can be a safe and feasible approach for the management of patients with tuberculous non-functional kidneys. However, further studies are necessary to confirm our results.

## References

[1] World Health Organization (2015). WHO fact sheet no. 104.

[2] Zarrabi AD, Heyns CF (2009). Clinical features of confirmed versus suspected urogenital tuberculosis in region with extremely high prevalence of pulmonar tuberculosis. Urology.

[3] Chibber PJ, Shah HN, Jain P (2005). Laparoscopic nephroureterectomy for tuberculous nonfunctioning kidneys compared with laparoscopic nephroureterectomy for other diseases. J Laparoendosc Adv Surg Tech A.

[4] Tian X, Wang M, Niu Y, Zhang J, Song L, Xing N (2015). Retroperitoneal Laparoscopic Nephroureterectomy for Tuberculous Nonfunctioning Kidneys: a single-center experience. Int Braz J Urol.

[5] Gupta NP, Hemal AK, Mishra S, Dogra PN, Kumar R (2008). Outcome of retroperitoneoscopic nephrectomy for benign nonfunctioning kidney: a single-center experience. J Endourol.

[6] Murray CJ, Ortblad KF, Guinovart C, Lim SS, Wolock TM, Roberts DA (2014). Global, regional, and national incidence and mortality for HIV, tuberculosis, and malaria during 19902013: a systematic analysis for the Global Burden of Disease Study 2013. Lancet.

[7] Global tuberculosis control: key findings from the December 2009 WHO report (2010). Wkly Epidemiol Rec.

[8] Cek M, Lenk S, Naber KG, Bishop MC, Johansen TE, Botto H (2005). EAU guidelines for the management of genitourinary tuberculosis. Eur Urol.

[9] Figueiredo AA, Lucon AM (2008). Urogenital tuberculosis: update and review of 8961 cases from the world literature. Rev Urol.

[10] Lee KS, Kim HH, Byun SS, Kwak C, Park K, Ahn H (2002). Laparoscopic nephrectomy for tuberculous nonfunctioning kidney: comparison with laparoscopic simple nephrectomy for other diseases. Urology.

[11] Zhang X, Zheng T, Ma X, Li HZ, Li LC, Wang SG (2005). Comparison of retroperitoneoscopic nephrectomy versus open approaches to nonfunctioning tuberculous kidneys: a report of 44 cases. J Urol.

[12] Modi P, Kadam G, Goel R (2007). Retroperitoneoscopic nephrectomy for pyonephrotic kidneys. J Endourol.

[13] Duarte RJ, Mitre AI, Chambô JL, Arap MA, Srougi M (2008). Laparoscopic nephrectomy outside gerota fascia for management of inflammatory kidney. J Endourol.

[14] Fang Z, Li L, Wang X, Chen W, Jia W, He F (2014). Total retroperitoneal laparoscopic nephroureterectomy with bladder-cuff resection for upper urinary tract transitional cell carcinoma. J Invest Surg.

[15] Miyazaki J, Nishiyama H, Fujimoto H, Ohyama C, Koie T, Hinotsu S (2016). Laparoscopic Versus Open Nephroureterectomy in Muscle-Invasive Upper Tract Urothelial Carcinoma: Subanalysis of the Multi-Institutional National Database of the Japanese Urological Association. J Endourol.

[16] Labanaris AP, Zugor V, Smiszek R, Nützel R, Kühn R (2010). Empyema of the ureteral stump. An unusual complication following nephrectomy. ScientificWorldJournal.

[17] Lai WR, Lee BR (2016). Techniques to resect the distal ureter in robotic/laparoscopic nephroureterectomy. Asian J Urol.

[18] Liu P, Fang D, Xiong G, Yang K, Zhang L, Yao L (2016). A Novel and Simple Modification for Management of Distal Ureter During Laparoscopic Nephroureterectomy Without Patient Repositioning: A Bulldog Clamp Technique and Description of Modified Port Placement. J Endourol.

[19] Phé V, Cussenot O, Bitker MO, Rouprêt M (2011). Does the surgical technique for management of the distal ureter influence the outcome after nephroureterectomy?. BJU Int.

[20] Shu-Xiong Z, Zhen-Sheng Z, Xiao-Wen Y, Hui-Zhen L, Xin L, Ying-Hao S (2014). Intraneobladder Hem-o-Lok Migration with Stone Formation after Orthotopic Neobladder Cystectomy. Case Rep Urol.

[21] Matin SF, Gill IS (2005). Recurrence and survival following laparoscopic radical nephroureterectomy with various forms of bladder cuff control. J Urol.

